# Sleep Characteristics of Parents of Children in the Paediatric Intensive Care Unit: A Pilot Observational Study

**DOI:** 10.1111/nicc.70609

**Published:** 2026-08-12

**Authors:** Hyejung Lee, Hyeryeong Lee, Sanghun Jung, Ai Soon Park

**Affiliations:** ^1^ Mo‐Im Kim Nursing Research Institute, College of Nursing Yonsei University Seoul South Korea; ^2^ College of Nursing The Ohio State University Columbus Ohio USA; ^3^ College of Nursing Yonsei University Seoul South Korea; ^4^ Severance Hospital Yonsei University Health System Seoul USA South Korea

**Keywords:** hospitalised child, paediatric intensive care unit, parents, sleep quality

## Abstract

**Background:**

Parents of children hospitalised in the paediatric intensive care unit (PICU) exhibit poor sleep quality due to stress from the child's illness, uncertainty and changing family roles.

**Aims:**

We aimed to utilise two subjective and one objective measurement to examine sleep characteristics among parents of children in the PICU and explore possible contributing factors, including parental stress.

**Study Design:**

This observational pilot study assessed parents of children in the PICU in South Korea using the Pittsburgh Sleep Quality Index (PSQI) and the General Sleep Disturbance Scale (GSDS). Sleep‐related parameters were measured for 3 nights using an actigraph. The Parental Stress Scale:PICU was used to assess stress levels.

**Results:**

A total of 18 parents participated in the study. Sleep quality was poor on both PSQI and GSDS, and their total sleep scores did not correlate with any actigraphy parameter. Only the GSDS daytime function subscale suggested associations with sleep efficiency (*p* = 0.04) and wake after sleep onset (*p* = 0.04). Parents averaged 5.73 h of sleep per night, with a sleep efficiency of 86.8%. The most prominent stressors were changes in parental roles and the child's appearance, while stress levels were not significantly related to sleep. No single factor influenced sleep across all tools; rather, different factors, including parent characteristics and length of stay, exerted an influence that varied by measurement tool. All participants completed the actigraphy protocol, proving its feasibility.

**Conclusions:**

Factors influencing parental sleep varied by measurement tool, suggesting that subjective and objective sleep measures may capture different aspects of sleep in parents. As this was a pilot study for hypothesis generation and feasibility, the findings require further validation in larger‐scale studies.

**Relevance to Clinical Practice:**

A comprehensive assessment that integrates objective and subjective sleep measures could improve the understanding of sleep among PICU parents and inform effective support strategies.

## Impact Statement

- What is known about the topic
  ○ Parents of children hospitalised in the PICU experience poor sleep quality, influenced by stress from their child's illness, uncertainty and changing family roles.
  ○ Actigraphy offers non‐invasive, objective sleep pattern measurements.
  ○ Using both subjective and objective methods can illustrate different sleep characteristics.
- What this paper adds
  ○ PICU parents reported changes in parental roles and the child's appearance as top stressors, and poor sleep quality, indicating a need for support.
  ○ Factors influencing parental sleep varied depending on the measurement tool, suggesting that subjective and objective sleep measures captured different aspects of parents' sleep.
  ○ All participants completed the actigraphy protocol, proving its feasibility. Interventions to support parental sleep can be developed using both types of measurements.

## 1 Introduction

Admission of a child to the paediatric intensive care unit (PICU) is a critical event that may adversely affect the physical, psychological and social well‐being of parents [1, 2, 3]. Parents often experience considerable stress related to their child's appearance during treatment, uncertainty about the child's condition, fear of death, parental role changes and the PICU environment [1, 2, 4, 5]. Having a family member in the intensive care unit (ICU) is a recognised contributor to poor sleep quality [6]. Parents of children in the PICU frequently report difficulty initiating sleep, frequent awakenings, daytime sleepiness and fatigue [7, 8, 9], which may contribute to anxiety, depression and stress [10, 11, 12]. Moreover, these adverse effects may persist after the child's discharge, negatively affecting parental health and quality of life [1, 13].

The family‐centred care model recognises parents as key decision‐makers who collaborate with healthcare providers to support their child's care and outcomes [14]. Accordingly, PICU nurses must identify factors that may impair parents' ability to fulfil this role. Poor sleep quality and sleepiness among family members of ICU patients contribute to impaired decision‐making, reduced cognitive functioning and difficulty performing daily activities [11, 15]. Moreover, interaction between poor sleep quality and elevated stress may exacerbate cognitive impairment [16]. These impairments may hinder parents' ability to understand medical information, navigate complex treatments and make critical decisions, underscoring the importance of monitoring parental stress and sleep quality. Previous studies have primarily assessed sleep in PICU parents using subjective measures, including self‐reported questionnaires and qualitative interviews [2, 7, 9]. These methods typically rely on recall over a defined period and may be less accurate in highly stressed populations.

Actigraphy provides an objective assessment of sleep patterns and multiple sleep parameters [8, 17]. As a small wrist‐worn device, it allows sleep monitoring in naturalistic settings with minimal disruption and shows good agreement with polysomnography, the gold standard for sleep assessment [17, 18]. However, few studies have used actigraphy to examine sleep patterns in parents of critically ill paediatric inpatients [3, 8]. This represents an important gap, as most research has relied on subjective approaches [2, 7, 9]. Subjective and objective assessments capture complementary dimensions of sleep quality [19, 20, 21], and their combined use may provide a more comprehensive understanding of sleep characteristics and associated factors in parents of children in the PICU.

## 2 Aims

This pilot study aimed to compare sleep characteristics in parents of children in the PICU using self‐report questionnaires and actigraphy data and to explore factors associated with sleep.

## 3 Design and Methods

### 3.1 Setting and Sample

This study used a cross‐sectional observational design. Participants were recruited from a 10‐bed PICU at a tertiary hospital in South Korea. We included parents of children who had been in the PICU for ≥ 3 days at the time, whose child remained in the unit during participation, and who were aged > 18 years. Only one parent per child participated. Participants were parents who visited the hospital to see their child and expressed interest in the study, regardless of primary caregiver status. Parents of children under a do‐not‐resuscitate order or those unable to understand Korean were excluded. As an exploratory pilot study, no formal sample size calculation was performed.

### 3.2 Variables and Instruments

#### 3.2.1 Demographic Characteristics

Data collected included parental age, sex, educational level, monthly income, number of children and sleeping location. Additionally, data were collected on the child's age, sex, primary diagnosis, PICU admission route, number of previous ICU admissions and current length of stay (LOS) in the PICU. The Paediatric Risk of Mortality III (PRISM III) score [22] was obtained from medical records to reflect the child's illness severity.

#### 3.2.2 Psychological Stress

The Korean version of the Parental Stress Scale:Pediatric Intensive Care Unit (PSS:PICU) was used to measure parents' stress levels [23]; it comprises 36 items across seven subcategories, rated on a five‐point Likert scale (1 = not at all stressful; 5 = extremely stressful) [24]. Items not experienced were scored as zero. The total score was calculated by dividing the sum of all item scores by the number of items scored ≥ 1 point. Content and construct validity have been established [24, 25], and the internal consistency has been reported [4, 24]; here, Cronbach's alpha was 0.93.

#### 3.2.3 Parental Sleep

Two self‐report questionnaires and actigraphy were used. The Korean version of the Pittsburgh Sleep Quality Index (PSQI) [26] was used to assess subjective sleep quality over the past month; it comprises 19 items across seven subcategories [27]. Higher scores indicate poorer sleep quality, and scores > 5 suggest significant sleep disturbance [27]. The validity and reliability of the Korean version of the PSQI have been demonstrated [26]. Cronbach's alpha of the Korean version of the PSQI was 0.84 [26], and here, it was 0.64.

The Korean version of the General Sleep Disturbance Scale (GSDS) was used to measure sleep disturbance [28]. It includes 21 items across six subscales regarding the frequency of each item experienced during the previous week (0–7 days) [28, 29]. The Diagnostic and Statistical Manual of Mental Disorders (DSM)‐IV criteria for insomnia were met when the mean total score or the mean of any of the three subscales was ≥ 3 [29]. The Cronbach's alpha reported for the original instrument was 0.88 [29], and in the current study, it was 0.81.

Actigraphy data (ActiGraph wGT3‐BT, Pensacola, FL), used to estimate sleep and wake patterns based on continuous movement counts, were collected at 30 Hz in 1‐min epochs. ActiLife version 6.13.4 software was used for the analysis, and the Cole–Kripke algorithm [17, 18, 30] was applied for sleep scores. In addition to wearing an actigraphy device, the research team sent participants daily text messages to verify actigraphy adherence from the previous night and to collect the bedtimes and wake times reported by participants to improve data accuracy. During data processing, participant‐reported bedtimes and wake times were incorporated into the actigraphy analysis to distinguish any off‐wrist periods from sleep and to estimate sleep parameters accurately.

For the statistical analysis, data on total sleep time (TST), sleep efficiency, wake after sleep onset (WASO) and the number of awakenings per night were collected over 3 days. TST represented total minutes scored as sleep, and sleep efficiency was calculated as TST divided by total time in bed. WASO was the total minutes scored as wake after sleep onset, and the number of awakenings per night was defined as the count of wake episodes after sleep onset and before wake times. Zak et al. [31] compared PSQI scores with actigraphy data obtained over 2 days, while Lau et al. [32] indicated that at least three nights of actigraphy are necessary for reliable estimation. Furthermore, given that the average LOS in the PICU was reported as 5–6 days [33, 34], a 3‐day actigraphy wearing period was chosen for feasibility.

### 3.3 Data Collection

Participants were primarily recruited through flyers posted in the PICU family waiting area. A research assistant discussed enrolment with parents who expressed interest in the study after screening them for eligibility. Data were collected between February 2023 and July 2024. Owing to the COVID‐19 pandemic, the hospital temporarily reduced visiting days from twice daily to once weekly; this was later eased to thrice weekly over the study period. Visitation was scheduled at noon, lasted 15 min, and parents were not allowed to enter the PICU outside this time.

After obtaining informed consent, parents completed the PSQI and provided demographic information. Subsequently, the research assistant instructed the parents to wear the actigraphy device during sleep for three consecutive nights and explained how to properly wear the device. Parents communicated their bedtimes and wake times via text messages. After 3 days of actigraphy monitoring, parents completed the GSDS and PSS:PICU. No pre‐sleep instructions or restrictions were imposed on participants. Although the three instruments had non‐coincident time frames, we retained each instrument's specified window to adhere to the scale's instructions. The PSQI and GSDS were administered on different days to minimise order effects of consecutive self‐report measures [35]. Following actigraphy monitoring, the GSDS was used to assess the number of days during the previous week, resulting in a partial overlap in the assessment period. All three instruments covered the child's hospitalisation period in the PICU, collectively providing insights into parental sleep.

If a participant failed to wear the actigraphy device on any night, they were instructed to wear it for an additional night to complete the 3‐day recording period. The children of all enrolled participants remained in the PICU for the study duration, and no participant withdrew from the study.

### 3.4 Data Analysis

**FIGURE 1:**
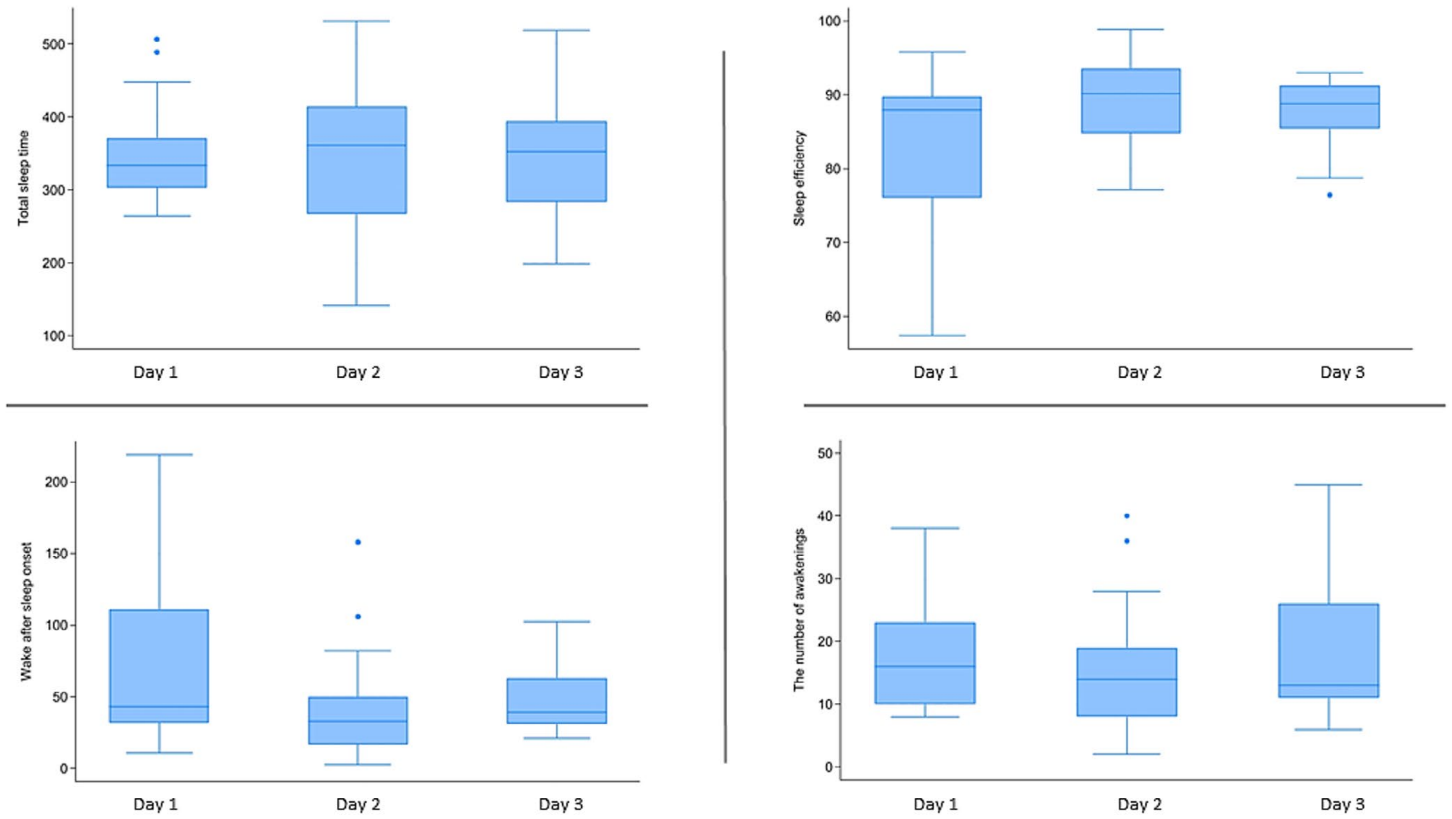
Distribution of actigraphy parameters across three nights. Actigraphy sleep parameters were analysed with 53 responses, which were from three nights of 18 participants.

Data were analysed using STATA/BE 18 (StataCorp LLC, College Station, TX). Descriptive statistics were calculated for demographic characteristics and PSS:PICU, PSQI, GSDS and actigraphy data. Spearman's rank correlation coefficient explored the relationship between sleep‐related variables. A multivariate regression analysed the relationship between subjective sleep quality and related factors. For actigraphy parameters, changes over the three‐night observation period were initially visualised (Figure 1). Given the absence of any consistent linear trends across parameters, multivariate regression was subsequently applied to explore factors related to actigraphy sleep parameters, with the mean value across three nights calculated for each patient and used as the dependent variable. The normality of regression residuals was assessed using Q‐Q plots. All statistical tests were two‐sided, with significance defined at alpha = 0.05, and 95% confidence intervals reported for relevant estimates.

### 3.5 Ethics Statement

This study was reviewed and approved by the Institutional Review Board of Severance Hospital, Yonsei University Health System (Study No. 4‐2022‐1465, approved on January 10, 2023). All participants provided written informed consent.

## 4 Results

### 4.1 Participant Characteristics

Of the 18 participants, 15 (83.3%) were mothers (mean age, 41.9 ± 5.29 years). Most participants had completed either high school (38.9%) or university (38.9%). The largest proportion (27.8%) reported a monthly income consistent with the national average [36]. Most parents (88.9%) slept at their usual residence (Table 1).

**TABLE 1:** Demographic characteristics of parents and children (*N* = 18).

Parent	Mean ± SD/*n* (%)	Child	Mean ± SD/*n* (%)
Age (years)	41.9 ± 5.29 (Range: 30–49)	Age (years)	9.2 ± 6.35 (Range: 1–17)
Mother	15 (83.3)	Gender	
Father	3 (16.7)	Boy	10 (55.6)
Education		Girl	8 (44.4)
High school	7 (38.9)	Number of siblings	
University	7 (38.9)	None	6 (33.3)
Graduate	4 (22.2)	1	9 (50.0)
Monthly household income (Korean won)		2	3 (16.7)
< 1 000 000	3 (16.7)	Admission route	
1 000 000 ~ 3 000 000	4 (22.2)	General ward	9 (50.0)
3 000 000 ~ 5 000 000	5 (27.8)	Operation room	2 (11.1)
5 000 000 ~ 7 000 000	3 (16.7)	Emergency department	3 (16.7)
≥ 7000,00	3 (16.7)	NICU	2 (11.1)
Place for sleep (*n*, %)		Other hospitals	2 (11.1)
Home (usual residence)	16 (88.9)	ICU admission experience	
Hospital	1 (5.6)	2	3 (16.7)
Other place	1 (5.6)	3	6 (33.3)
		4	5 (27.8)
		≥ 5	4 (22.2)
		Length of PICU stay	13.1 ± 10.37 (Range: 5–50)
		PRISM III score	4.3 ± 6.13 (Range: 0–25)

Abbreviations: NICU, Neonatal Intensive Care Unit; PICU, Paediatric Intensive Care Unit; PRISM III, Paediatric Risk of Mortality III; SD, standard deviation.

The children's median age was 9.2 ± 6.35 years; 10 were boys (55.6%). Twelve children (66.7%) had one or more siblings. The PICU admission route was primarily from the ward (50.0%). All children had a history of PICU/neonatal intensive care unit (NICU) admission; half were admitted to the ICU ≥ 4 times, including the current admission. The median PICU LOS at participation was 13.1 ± 10.37 days. The median PRISM III score at PICU admission was 4.3 ± 6.13 (Table 1).

### 4.2 Parental Stress

The mean parental stress score was 3.3 ± 0.44. Parents reported the highest stress scores related to their parental role (mean 3.9 ± 0.65). The lowest stress score was reported for professional staff behaviour (mean 2.7 ± 0.84) (Table 2).

**TABLE 2:** Descriptive statistics of major variables (*N* = 18).

PSS:PICU	Mean ± SD
Total score	3.3 ± 0.44
Behaviours and emotional response	3.2 ± 0.60
Parental roles	3.9 ± 0.65
Procedures	3.3 ± 0.44
Sights and sounds	3.1 ± 0.96
How professional staff communicate	3.2 ± 0.56
Behaviours of the professional staff	2.7 ± 0.84
Child's appearance	3.6 ± 0.45

PSQI	Mean ± SD or *n* (%)
Global score	9.2 ± 3.29 (range: 6–17)
Global score > 5^a^	15 (100)
Subjective sleep quality	1.8 ± 0.79
Sleep latency	2.0 ± 0.84
Sleep duration	1.2 ± 1.20
Habitual sleep efficiency	0.7 ± 1.07
Sleep disturbance	1.5 ± 0.62
Use of a sleeping medication	0
Daytime dysfunction	1.9 ± 0.87

GSDS	Mean ± SD or *n* (%)
Average total score	2.4 ± 0.88 (range: 1.6–4.6)
Average total score ≥ 3^b^	4 (22.2)
Any mean of 3 subscales ≥ 3^b^	14 (77.8)
Initiation insomnia	4.1 ± 2.07
Maintenance insomnia	3.7 ± 2.19
Sleep quality	4.0 ± 1.51
Sleep quantity	2.1 ± 1.46
Daytime function	3.3 ± 1.37
Medication/self‐help for sleep	0.1 ± 0.36

**Actigraphy** ^**c**^	**Mean ± SD**
Total sleep time (min)	343.8 ± 85.77 (range: 141–532)
Sleep efficiency (%)	86.8 ± 7.49 (range: 57.36–98.89)
Wake after sleep onset (min)	53.6 ± 41.87 (range: 3–219)
Awakenings (per night)	17.2 ± 9.84 (range: 2–45)

Abbreviations: GSDS, General Sleep Disturbance Scale; PSQI, Pittsburgh Sleep Quality Index; PSS:PICU, Parental Stress Scale:Paediatric Intensive Care Unit; SD, standard deviation.

^a^ A PSQI global score greater than 5 points indicates significant sleep disturbance.

^b^ A GSDS average total score or the mean of any of the three subscales greater than 3 is considered to meet the Diagnostic and Statistical Manual of Mental Disorders (DSM)‐IV criteria for insomnia.

^c^ Actigraphic parameters were analysed with 53 responses, which were from three nights of 18 participants.

### 4.3 Sleep Characteristics of Parents

The mean global PSQI score was 9.2 ± 3.29, with all participants reporting a global score > 5, indicating poor sleep [27]. The mean GSDS total score was 2.4 ± 0.88, and 14 parents (77.8%) had an average score of ≥ 3 on any three subscales, meeting the criteria for insomnia [29] (Table 2). No participant reported using sleep medication on the PSQI. On the GSDS, two participants reported using alcoholic beverages and/or pain medication for sleep. In the PSQI free‐text responses on additional sleep disturbances, childcare‐related disruption was reported by four participants, noise by one, anxiety by one and physical pain/discomfort by two.

Based on 3 nights of actigraphy (53 nights after excluding one night for poor data quality), the average TST was 343.8 ± 85.77 min; sleep efficiency was 86.8% ± 7.49%. Mean WASO was 53.6 ± 41.87 min, and sleep awakenings were 17.2 ± 9.84 per night. One night of data from one participant was excluded due to incorrect device placement (Table 2).

Correlational analysis revealed no significant associations between actigraphy parameters and either the PSQI global score or the GSDS average total score (Supplementary S1). Among PSQI and GSDS subscales, only the GSDS daytime function subscale was positively and negatively associated with sleep efficiency (*r*_s_ = 0.4922, *p* = 0.04) and WASO (*r*_s_ = −0.4951, *p* = 0.04), respectively (Table 3).

**TABLE 3:** Correlations between subjective and objective sleep‐related variables (*N* = 18).

Actigraphic parameters	PSQI global score	GSDS average total score	GSDS daytime function
Total sleep time^a^	−0.1100	−0.2587	−0.2477
Sleep efficiency^a^	−0.3311	0.2442	0.4922*
Wake after sleep onset^a^	0.1782	−0.2205	−0.4951*
Awakenings^a^	0.2118	−0.1289	−0.3437

Abbreviations: GSDS, General Sleep Disturbance Scale; PSQI, Pittsburgh Sleep Quality Index; SE, sleep efficiency; WASO, wake after sleep onset.

^a^ The mean of three nights' actigraphy parameters was used in the analysis.

* *p* < 0.05.

LOS (*p* = 0.01) and monthly household income (*p* = 0.005) correlated with sleep, with higher PSQI global scores observed among parents whose child had a shorter PICU stay and who reported lower income. Regarding the GSDS average total score, parents with a university‐level education or higher were more likely to report higher scores than those with only a high school education (*p* = 0.03) (Table 4). In the multivariate regression models with actigraphy parameters as dependent variables, including TST, sleep efficiency, WASO and the number of awakenings per night, none of the included independent variables showed any statistically significant associations (Table 4).

**TABLE 4:** Factors associated with sleep quality across three different measurements (*N* = 18).

		PSQI score (95% CI)	GSDS score (95% CI)	Actigraphy parameters (95% CI)			
				TST^a^	SE^a^	WASO^a^	Awakenings^a^
Parental stress		−1.94 (−6.28, 2.39)	0.86 (−0.28, 2.00)	48.17 (−69.99, 166.34)	1.20 (−10.43, 12.83)	1.39 (−66.63, 69.41)	5.78 (−12.41, 23.98)
Length of stay		−0.26** (−0.44, −0.08)	−0.03 (−0.08, 0.02)	2.37 (−2.60, 7.33)	−0.30 (−0.78, 0.19)	1.81 (−1.04, 4.67)	0.31 (−0.46, 1.07)
Child age		−0.04 (−0.34, 0.25)	0.06 (−0.01, 0.14)	0.70 (−7.40, 8.80)	0.42 (−0.38, 1.22)	−2.19 (−6.85, 2.47)	−0.54 (−1.79, 0.70)
PRISM III		−0.05 (−0.38, 0.29)	−0.03 (−0.12, 0.05)	−2.34 (−11.40, 6.72)	0.04 (−0.85, 0.93)	−0.76 (−5.98, 4.46)	−0.20 (−1.60, 1.19)
Admission route	Non‐general wards^b^	1.29 (−2.09, 4.66)	0.04 (−0.85, 0.93)	64.27 (−27.84, 156.39)	5.36 (−3.71, 14.42)	−15.55 (−68.58, 37.47)	−2.14 (−16.32, 12.04)
Previous admission	> 2^c^	−2.21 (−7.30, 2.88)	−0.48 (−1.81, 0.86)	122.84 (−15.94, 261.63)	−3.03 (−16.68, 10.63)	26.45 (−53.44, 106.34)	7.56 (−13.81, 28.93)
Monthly income	≥ 5 million Korean Won^d^	−5.32** (−8.60, −2.04)	−0.70 (−1.56, 0.16)	61.57 (−27.78, 150.91)	1.64 (−7.15, 10.43)	−2.64 (−54.07, 48.79)	1.37 (−12.39, 15.13)
Education	University or higher^e^	3.59 (−0.02, 7.20)	1.06* (0.12, 2.01)	9.99 (−88.33, 108.32)	4.35 (−5.33, 14.02)	−18.04 (−74.64, 38.56)	−6.25 (−21.39, 8.89)

*Note:* Values represent coefficients.

Abbreviations: CI, confidence intervals; GSDS, General Sleep Disturbance Scale; PRISM III, Paediatric Risk of Mortality III; PSQI, Pittsburgh Sleep Quality Index; SE, sleep efficiency; TST, total sleep time; WASO, wake after sleep onset.

^a^ The mean of three nights' actigraphy parameters was used in the analysis.

^b^ Reference group: General ward.

^c^ Reference group: ≤ 2.

^d^ Reference group: < 5 million Korean won.

^e^ Reference group: High school.

* *p* < 0.05.

** *p* < 0.01.

### 4.4 Actigraphy Feasibility

Overall, 19 participants were recruited; one withdrew because of discomfort when wearing the actigraphy device before data collection. Among the remaining 18 parents, three forgot to wear the device for three consecutive nights, requiring an additional night of monitoring. One participant stayed up all night without sleeping during the study; therefore, she wore the device for an additional night. No participant reported problems while wearing the device.

## 5 Discussion

Our use of both subjective and objective measures contributed to the current knowledge by employing a multi‐method approach to triangulate findings, which is rare in PICU parents. It allowed for a comprehensive assessment, suggesting that different tools capture distinct aspects of sleep. Moreover, this study supported the feasibility of employing multiple sleep measures in this population.

Most parents reported significant sleep disturbances and clinically meaningful insomnia, as measured by the PSQI and GSDS, respectively. Additionally, parents showed insufficient sleep quantity based on actigraphy, as the mean TST (5.73 h) was lower than the minimum sleep duration recommended for healthy adults [37]. Sleep duration was relatively short; however, sleep efficiency was similar to that reported in a previous study of PICU parents in Canada [8]. Compared with parents of children in the general wards in Sweden, the United Kingdom and the United States, those in this study reported a relatively shorter TST but higher sleep efficiency [38, 39, 40], potentially related to the complex dynamics of the child's critical condition and the sleeping environment of the parents during PICU admission across studies. Stremler et al. [8] reported that parents were allowed to stay overnight in the PICU with their child and were provided with fold‐out beds, suggesting that bedside presence may affect parental sleep. Children in general wards tend to have better health conditions than those in specialised units such as the PICU or NICU, and sleep disturbance related to the child's severity of illness is relatively lower. However, parents may still experience poor sleep patterns due to continuous caregiving and communication demands during inpatient care. Herein, parents were not permitted to stay at their child's bedside during the night, particularly during and after the COVID‐19 pandemic period. Therefore, the results should be interpreted and compared with caution.

Among parental stressors, role alteration was the most prominent, followed by changes in the child's appearance, consistent with previous studies from Croatia and India and findings from a systematic review [1, 4, 5]. During the data collection period, parents were allowed to visit only 1–3 times per week, as the PICU had stricter visiting policies than before. Notably, restrictive visitation policies influence psychological stress and burdens in the families of patients admitted to the ICU, as found in studies from South Korea and a scoping review [6, 41]. The limitation of parental presence can significantly reduce opportunities for parents to participate in the decision‐making process [42], resulting in changes to their perceived parental roles.

Neither the PSQI global score nor the GSDS total score was associated with any actigraphy parameters, except for associations between a GSDS subcategory and the sleep efficiency and WASO parameters. Previous studies from the United States, France and the United Kingdom have likewise reported inconsistent or weak correlations between subjective sleep quality and actigraphy‐derived parameters [31, 43, 44], potentially reflecting the possibility that each captured distinct aspects of sleep and the different time windows used across the three measurements. As subjective sleep measures, the PSQI primarily assesses sleep disturbances over the previous month, whereas the GSDS focuses on insomnia, capturing related but distinct aspects of sleep. Moreover, both long and short TST can indicate poor subjective sleep quality [45], highlighting the complementary roles of subjective and objective measures for improved understanding of sleep characteristics. Additionally, subjective sleep quality can be influenced by various factors that shape individuals' perceptions of their sleep [44]. Negative emotions such as anxiety or depression may affect an individual's perception of sleep quality, while showing little association with objective sleep patterns [44]. Similarly, in the present study, sleep was assessed among PICU parents experiencing the highly stressful situation of having a critically ill child; their unmeasured negative emotions may have influenced subjective sleep, resulting in inconsistencies or a lack of association with objective sleep parameters.

Despite our initial expectations, parental stress was not significantly associated with sleep, as measured by any of the three instruments. This may be explained by the stress measure employed, which assesses stress during a child's PICU stay [24] and may not adequately capture chronic parental stress. Notably, the children in our study were admitted to the PICU more than twice, suggesting ongoing health‐related challenges and prolonged parental chronic stress exposure. Thus, sleep may have already been influenced by chronic stress, making the effects of acute stress related to the current PICU hospitalisation less noticeable.

Interestingly, no single parent or child characteristic influenced all three sleep measurements simultaneously; associated factors varied by measurement. Therefore, sleep assessment may differ by tool, adding complexity to the interpretation and comparison of intervention effects on sleep outcomes in clinical studies. Applying multiple methods may be more beneficial than relying on a single approach because it enables a more comprehensive assessment of the various dimensions of sleep and relevant factors [19, 20, 21, 44].

Longer PICU stay was associated with better subjective sleep quality among parents. Spending more nights in the PICU significantly contributed to poor sleep among PICU parents in previous research from Spain [7], which was not clearly reflected in either subjective measures or actigraphy sleep data in this study. The association of a longer LOS with better subjective sleep quality may partially reflect the institution's visitation policy, which limits parental presence in the PICU. This policy may have allowed parents to rely on the PICU nursing team while sleeping at home without providing direct physical care for their child, thereby positively affecting their sleep. Moreover, our results suggest that parents become more familiar with the PICU environment and critical care processes during prolonged admissions. Here, all children had LOS ≥ 5 days, with a mean of 13 days at the time of study participation. This familiarity may help them develop coping strategies for managing their child's illness [46], which may positively affect their sleep. However, these interpretations require further investigation, as these factors were associated with only one subjective sleep measurement, and our findings cannot be directly compared with the sleep of parents throughout the early hospitalisation phase.

No participants withdrew during the actigraphy period, except one parent who withdrew before data collection due to discomfort during trial wear. This finding suggests high adherence to the research protocol, supporting the feasibility of using the device in this population. Although all participants completed three nights of actigraphy measurements, some did not wear the device for three consecutive nights. Therefore, evening reminders could help participants wear the device before bed and improve data quality. Additionally, although actigraphy was applied for the minimally recommended duration of 3 nights in the present study, longer monitoring periods may allow for a more accurate capture of sleep patterns and variability [32]. Given the feasibility demonstrated here, future studies can extend the duration of actigraphy monitoring to provide a more comprehensive understanding of sleep characteristics in this population.

## 6 Limitations

First, this exploratory pilot study involved a small sample, which limited statistical power and introduced potential selection bias. Additionally, due to the predominance of mothers in the study sample and the absence of a comparison group, the results should be interpreted with caution due to the limited generalisability to all PICU parents. Second, the study began during the COVID‐19 pandemic, when hospital visiting policies were highly restrictive and gradually relaxed over time; these changes may have confounded the results by affecting parental stress and sleep patterns. Third, having different time windows for the three sleep measurements made it difficult to compare the results directly.

## 7 Implications for Practice and Further Research

Given this study's pilot and exploratory nature, the findings primarily serve to generate hypotheses for future research, which should validate the results and investigate underlying mechanisms by involving a larger sample of PICU parents from multiple centres, greater variation in parent and child characteristics and longer actigraphy monitoring periods. Additionally, longitudinal and interventional studies are needed to examine changes in parental sleep over time and their impact on decision‐making in PICUs. In practice, nurses could play a key role in monitoring parental sleep quality and providing appropriate support based on identified potential stressors in the PICU, with implications for parental decision‐making and roles.

## 8 Conclusions

Our study enhances the understanding of sleep quality among parents of children admitted to the PICU and suggests the need for multiple assessments. Poor sleep quality reported by PICU parents highlights the need for nurses to monitor their sleep status with relevant stressors and provide appropriate support, as it may negatively affect decision‐making and parental roles. Furthermore, employing both objective and subjective sleep measurements could be useful for capturing different dimensions of sleep quality, providing valuable insights for supporting these parents.

## Author Contributions

Hyejung Lee: conceptualisation, funding acquisition, methodology, project administration, resources, supervision, validation, visualisation, writing – review and editing. Hyeryeong Lee: conceptualisation, data curation, formal analysis, funding acquisition, investigation, methodology, resources, validation, visualisation, writing – original draft, writing – review and editing. Sanghun Jung: resources, writing‐review and editing. Ai Soon Park: resources, writing‐review and editing.

## Funding

This work was supported by a 2023 faculty–student research grant from the Yonsei University College of Nursing.

## Ethics Statement

This study was reviewed and approved by the Institutional Review Board of Severance Hospital, Yonsei University Health System (Study No. 4‐2022‐1465, approved on January 10, 2023). The study was conducted per the ethical standards of the responsible institutional review board and in accordance with the Helsinki Declaration of 1975. All participants were informed of the purpose of the study and their right to withdraw voluntarily and completed the consent form before participating.

## Consent

All participants provided informed consent prior to participation in the study.

## Conflicts of Interest

The authors declare no conflicts of interest.

## Supporting information


**Supplementary S1** Scatter plots of PSQI and GSDS scores in relation to actigraphy sleep parameters.

## Data Availability

The data that support the findings of this study are available on request from the corresponding author. The data are not publicly available due to privacy or ethical restrictions.
