# Supplementary material for: Sleep Characteristics of Parents of Children in the Paediatric Intensive Care Unit: A Pilot Observational Study
Source: Nurs Crit Care. 2026 Aug 12;31(5):e70609. doi: 10.1111/nicc.70609 (PMC13463211; doi:10.1111/nicc.70609)
Supplement: Supplementary file 1 [file NICC-31-0-s001.docx]

**Supplementary 1**.

Scatter Plots of PSQI and GSDS Scores in Relation to Actigraphy Sleep Parameters

*Note.* Awakening = the number of awakenings per night; GSDS = General Sleep Disturbance Scale; PSQI = Pittsburgh Sleep Quality Index.

The left panel (blue dots) represents the association between PSQI subscales & global score and actigraphy sleep parameters. The right panel (orange dots) represents the association between GSDS subscales & total average score and actigraphy sleep parameters.
